# Alcohol wipes are the most justifiably sustainable disinfection method for Goldmann tonometry

**DOI:** 10.1038/s41433-025-03855-6

**Published:** 2025-05-20

**Authors:** James W. Corbett, Sukhpal S. Sandhu, Jesse Gale

**Affiliations:** 1 Wellington Regional Hospital, Te Whatu Ora Health New Zealand, Wellington, New Zealand; 2 The Royal Victorian Eye and Ear Hospital, Melbourne, VIC Australia; 3 University of Melbourne, Melbourne, VIC Australia; 4 Centre for Eye Research Australia, Melbourne, VIC Australia; 5 University of Otago Wellington, Wellington, New Zealand

**Keywords:** Health care economics, Epidemiology

## Abstract

The essential technique of Goldmann tonometry is thought to have a risk of nosocomial infection, and thus there has been pressure to use single-use tonometer tips or slower and more-costly methods of disinfection for reusable tips. Here we review the evidence of infection from tonometry and the evidence behind different disinfection methods. The only infection reported to be transmitted by tonometry is adenoviral epidemic keratoconjunctivitis, which is also transmitted without tonometry and is a relatively low-morbidity infection. More serious eye infections or systemic infections have never been reported to be attributed to tonometry or eye examination. The most popular, affordable, quick and sustainable method of disinfection, using alcohol swabs to wipe the tip, is the only economically justifiable method.

## Introduction

Goldmann applanation tonometry (GAT) is the gold standard for intraocular pressure measurement, an indispensable technique, estimated to be performed over 120 million times per year [1]. Applanation tonometry requires contact between the corneal surface and the tonometer tip, which raises concern for the possibility of nosocomial transmission of infections from GAT. All clinical care involves contact between people and surfaces such as chairs, cutlery, door handles, so therefore, pragmatic, targeted, evidence-based and sustainable use of disinfection is required. Here we review the evidence of the infection risk from the eye clinic, including in a large Australian eye hospital, and the justification for different disinfection methods.

This was a narrative review. We performed an electronic database search including Cochrane Library, PubMed and Google Scholar with keywords including tonometry or tonometer and iatrogenic, nosocomial, ocular or systemic infection, and reviewed search results that were in English.

## Tonometer-associated ocular infections

A wide variety of infections could theoretically be spread by GAT: systemic infections such as variant Creutzfeld Jakob disease or human immunodeficiency virus, bacterial or fungal keratitis, or ocular viral infections such as herpes simplex or adenoviral keratoconjunctivitis [2]. Viral proteins have been detected in the tear film of infectious people, and transmission of hepatitis B via inoculation of the ocular surface was demonstrated in a chimpanzee [3]. However, in our review of available literature, after decades of GAT with reusable tips (several billion examinations), we did not identify a single reported case of microbial keratitis or systemic infection suspected to be contracted from tonometry.

We examined the reporting system for hazard identification at The Royal Victorian Eye and Ear Hospital in Melbourne, Australia between January 2015 and January 2024. In that period there were 253,405 presentations to the eye casualty service, over 900,000 attendances at eye clinics, and zero reports of keratitis (or other infection) that were suspected to relate to tonometry. In Australia and New Zealand, where the standard method of tonometer disinfection has been wiping with 70% isopropyl alcohol, tonometry and eye clinic attendance are not recognised as risk factors for serious eye infections [4–7].

The only infection implicated in nosocomial spread by tonometry is adenoviral epidemic keratoconjunctivitis (EKC) [8, 9]. Human adenovirus is a non-enveloped virus that binds CD46 on the ocular surface, promoting internalisation of the virus into epithelial cells, and the protein capsid resists degradation by alcohols [10]. Early reports of EKC recognised that spread occurs from fomites, in families, and at workplaces, and while aspects of ophthalmic examination have been suspected causes of transmission, no direct evidence has linked tonometry to EKC (Table 1) [11].

**Table 1:** Nosocomial outbreaks of epidemic keratoconjunctivitis.

Country	Year^a^	Cases (*n*)	Viral Pathogens	Cases with prior eye clinic visit (*n*, %, clinic staff affected)		Cases who had tonometry (*n*, %)		Reference
USA	1968	44	HAdV-8	16	36%	21	48%	[51]^b^
England	1971	45	HAdV-8	30	66% (3 staff)	15	33%	[52]
Canada	1974	20	HAdV-19	17	85%	16	80%	[53]^c^
England	1977	19	HAdV- 10, −19	3	15%			[54]
England	1978	113	HAdV-4	9	8% (1 staff)	0	0	[55]
England	1982	200	HAdV-8	132	66%	7	3.5%	[56]
England	1983	186	HAdV-8	25	13% (2 staff)	0	0	[57]
USA	1986	132	HAdV-8		3 staff			[58]^d^
Germany	1991	145	HAdV-8	44	30%	14	32%	[59]
Canada	1995	38	HAdV-8 (D)					[60]^e^
England	1999	38	HAdV-1, −2, −3, −5, 6	19	50% (4 staff)	7	47%	[61]
Spain	1999	266	HAdV	46	17% (23 staff)	22	48%	[62]
Spain	2005	46	HAdV-8	0	0%	0	0%	[63]
Chile	2006	681	HAdV	150	29%			[64]
Japan	2007	27	HAdV-3, −7	24	88% (3 staff)			[65]^f^
USA	2008	70	HAdV-8	33	47% (8 staff)			[9]
USA	2009	37	HAdV-8	23	26% (1 staff)			[9]
USA	2009	18	HAdV-19	16	89% (4 staff)	0	0%	[9]
USA	2010	286	HAdV-8, −3	156	55%(1 staff)			[9]
England	2019	126	HAdV-D8	0	0% (5 staff)	0	0%	[66]^g^

^a^Year of publication if not specified.

^b^No Goldmann disinfection used.

^c^Single Shiotz tonometer with no disinfection.

^d^Single pneumotonometer with no disinfection.

^e^Unclear what proportion of outbreak was transmitted in eye clinic, tonometry not associated.

^f^Outbreak in inpatient eye ward, HAdV-3 found on tonometer tip was only cultured from one of 27 cases.

^g^Only staff cases were suspected to have been possibly contracted in the clinic, all other cases were contracted in the community. Eye clinic staff infections are relevant as they are contracted in the eye clinic but they do not undergo tonometry, and most staff do not perform tonometry.

These reports of EKC outbreaks highlight that adenovirus is often spread without tonometry, in communities and in the eye clinic, and most clinics in these reports have found hand washing and regular wiping of clinic surfaces to be effective in eliminating these outbreaks.

Epidemic keratoconjunctivitis is the one reported nosocomial eye infection from the clinic (not contracted from surgery or injections). Other examples of nosocomial eye infections include bacterial keratitis associated with intensive care units (ICU), or neonatal intensive care but none of these are reported to be contracted from eye clinics or eye examinations [12–15]. Methicillin-resistant *Staphylococcus aureus* (MRSA) has been implicated as a nosocomial agent capable of causing ocular infection, and is more prevalent in long-term care facilities, neonatal ICU and nurseries. In a large retrospective review of 3640 MRSA infections, 49 (1.3%) were eye-related, of which 12 were identified as nosocomial. These hospital-acquired infections included four eyelid and periorbital infections, which developed as complications after eyelid or facial surgery. Four cases of neonatal conjunctivitis were classified as nosocomial. Three people with MRSA corneal ulcers were hospital acquired, one had an allergic shield ulcer that failed to improve with topical ciprofloxacin, another developed keratitis after a traumatic and chemical injury became infected, and the other had both ophthalmic shingles and was in hospital after neurosurgery when their infection developed. There was no association with previous visits to the eye clinic or any examination procedures like tonometry [16].

## Tonometer tip disinfection

Despite this limited evidence that tonometry is a risk for adenovirus transmission, there is a body of literature on how to disinfect tonometer tips between uses. The effectivity of disinfectants is based on experimental and in vitro studies but not on epidemiological or clinical evidence. While bacterial contamination is relatively easy to eliminate [17], there is some conflicting evidence that adenovirus is better eradicated by soaking in bleaches than alcohols [18–21]. A potential risk of transmission of variant Creutzfeld Jakob Disease was shown experimentally [22], but no disinfection method has been shown to eliminate protein from a reusable tonometer tip [23, 24]. Systematic reviews have found no definitive conclusion as to the optimal method for disinfection [2, 25].

Practically, the disinfection techniques in use are 1) wiping the tip with alcohol, usually 70% isopropyl alcohol, which also removes particulate contamination, then allowing time to dry, or 2) soaking the tip in a specific concentration of disinfectant for a minimum duration, before rinsing and drying. Soaking methods consume more time and staff resources, require a number of tips in circulation, accelerate the degradation of tips, and may lead to chemical injuries if rinsing is insufficient. There is also a theoretical risk of growing and transmitting resistant organisms between tips if the soaking solution is reused for long periods. The third option, single-use disposable tips is the most costly option, but even these are may be handled with fingers before use, or can be re-used inadvertently, so they do not eliminate the theoretical risk of infection [1, 26]. Concerns have been raised about contamination of the holders of these single-use disposable tips, too [27]. Disposable tips substantially increase the costs and associated carbon footprint of tonometry [1, 28–30].

In Australia and New Zealand, isopropyl alcohol wipes remain the most popular technique, common in Canada and USA surveys too [1, 21, 31]. In a 2008 survey of British eye departments, 77% used reusable tips, and these were disinfected using sodium hypochlorite (67%), isopropyl alcohol wipes (15%) or hydrogen peroxide (10%) [32]. (The few other departments used chloramine, chlorhexidine or peracetic acid solutions, all of which have the same requirement for rinsing and drying) [32].

## The mechanism of common disinfectants

### Alcohols

Alcohols provide disinfectant action against a wide spectrum of microorganisms by denaturing proteins, however, do not inactivate spores and do not effectively penetrate protein-rich materials. Ethyl alcohol (ethanol) is a useful disinfectant but is more tightly regulated due to the potential for misuse. 70% isopropyl alcohol has been shown to be ineffective against poliovirus and adenovirus as a soaking solution, as these non-enveloped viruses have a dense protein capsid [33, 34]. Using alcohol wipes can result in keratopathy if the tip is not dry or if swabs with additional disinfectants such as chlorhexidine are used without rinsing [35]. It is hard to quantify the morbidity caused by this but it has led some institutions to stop using alcohol disinfection (personal communication).

### Chlorine compounds

Dilute bleach is commonly used as a disinfectant in the healthcare setting. Chlorine compounds denature proteins and oxidise cell membranes, leading to effectivity against bacteria, enveloped and non-enveloped viruses, mycoplasma, mycobacterium and fungi. Chlorine-based solutions should achieve a concentration of >5000 ppm (in accordance with the CDC and AAO recommendations for HIV inactivation) [36, 37].

### Hydrogen peroxide

Free radicals produced by hydrogen peroxide-containing cleaning products oxidise cell membranes and critical intracellular components such as DNA. 10% hydrogen peroxide has been shown to be effective against a wide range of organisms including bacteria, enveloped and non-enveloped viruses, fungi, yeasts, bacteria and acanthamoeba cysts. Hydrogen peroxide carries the risk of significant corneal toxicity if not removed from the tonometer tip after disinfection [38].

Related to the choice of disinfectant is the issue of degradation of the tip, because after multiple cycles of disinfection the surface of a reusable tip can become porous or scratched, increasing the theoretical risk of harbouring infectious particles between cases. Immersion in isopropyl alcohol, or repeated simulated wiping was noted to reduce the lifespan of the tip to 1–2 years [39, 40]. Structural damage has been found on Schiotz tonometers with a 1:10 concentration of sodium hypochlorite (5000 ppm chlorine) and 3% hydrogen peroxide [41].

## Existing guidance on disinfecting tonometer tips

There is no international standard or consensus on tonometer tip disinfection. The CDC currently classes tonometer tips as semi-critical devices and recommends soaking in either 5000 ppm chlorine or 70% ethyl alcohol for 5–10 min, followed by a thorough tap water rinse and air drying [36]. The American Academy of Ophthalmology (AAO) recommends soaking tips in 1:10 sodium hypochlorite because it is thought to be more effective as a virucidal agent than isopropyl or ethyl alcohol. This guideline highlights the need to regularly inspect tips for damage or degradation and recommends single-use tips for patients with suspected prion disease [37].

Despite this guidance, a member survey was sought by the AAO in collaboration with the American Glaucoma Society (AGS) to ascertain current trends in tonometer preference, tonometer use, disinfection process, disinfectants, disinfection timing, and tonometer damage [31]. The survey revealed that the use of reusable Goldmann tonometer tips was widespread (79% of AAO and 45% AGS), and that most ophthalmologists clean with 70% isopropyl alcohol wipes (77% AAO and 61% AGS).

The Royal College of Ophthalmologists in the UK recommends the use of non-contact tonometry or single-use disposable tips and probes in most situations, and recommends soaking reusable tips in sodium hypochlorite 1% for 10 min. Their guidance emphasises that reusable tips should not be allowed to dry after use because prions can bind tightly to the dried surface of the tip [42].

The Royal Australian and New Zealand College of Ophthalmologists has a guideline for preventing nosocomial spread of EKC, and in the context of an outbreak or potentially infected patients this recommends single-use disposable tips or probes [43]. Their guideline on routine tonometry argues that alcohol wipes are sufficient given the very high cost to prevent mild infections [44].

Tonometer tip manufacturers provide a table of branded disinfection products that have good material compatibility with their polymethyl methacrylate (PMMA) tips, however, the manufacturers of the tips and disinfection products do not test the products for microbiological efficacy in the context of tonometry Appendix 1. shows the limitations of microbial efficacy data obtained from disinfection product manuals or, where indicated, external research. The reusable PMMA tips have been reported to be degraded by alcohols [45–47], including opacification and degradation of the glue, and this is the reason they are not recommended by the tip manufacturer. The manufacturers also recommend replacement of tonometer tips after 2 years, after 100 disinfections, or if visibly damaged. We revisited this issue by simulating two years of regular tip use with > 7680 wipes or 25 days of constant soaking with 70% isopropyl alcohol (much more exposure than previous studies) and found no detectable damage to the reusable PMMA tip [48]. Likewise, a reusable tip in constant use for many years, with an estimated >30,000 wipes of 70% isopropyl alcohol, showed no evident deterioration of the glue or clinically significant changes in clarity or performance [48].

## Cost effectiveness of disinfection methods

As the morbidity attributed to tonometry-related infections has been limited to adenoviral EKC, it has not been possible to describe the number of quality adjusted life years (QALYs) that are saved through different infection control measures. One pivotal study estimated the cost associated with trying to prevent one case of tonometer-associated EKC, using alcohol wipes or peroxide soak disinfection methods, or single-use disposable tips [1]. This group estimated the additional time and resources of soaking, and the additional costs of single-use tips, the potential reductions in transmission of adenovirus with soaking or disposable tips, and modelled the risk of nosocomial transmission with a community baseline risk of EKC. The incremental cost effectiveness ratio (ICER) was calculated at CAD $12,000 for peroxide to prevent one case of EKC, and CAD $51,000 for single-use tips to prevent one case of EKC [1]. When considering only more severe cases with corneal involvement affecting vision, The ICER were CAD $122,000 for peroxide and CAD $613,000 for single-use tips. When the background prevalence of EKC was reduced, the ICER were CAD $304,000 for peroxide and CAD $1,500,000 for single-use tips. Even in models where half of all EKC cases were transmitted in hospital eye clinics, the ICER was over CAD $12,000 for peroxide to prevent one case with vision threatening complications. These high costs, to prevent a relatively low-morbidity infection, are not justifiable or sustainable. An analysis in 2017 suggested around £2 million could be saved in the United Kingdom every year if reusable tonometry tips were used, while being safe and reliable [30]. This represents a £2 million opportunity cost if the money spent on disposable tips were put towards more effective, higher-value health care.

The impact of wasteful health spending on low-value care is also important in the context of sustainability. While most of the climate change effects of glaucoma care are emissions from patient and staff transport [49], there is a major contribution to ophthalmology emissions from the production and supply of single use disposables [50]. While the embedded emissions in the production and supply of tonometer tips are not publicly available, single-use tips certainly represent a large volume of plastic waste [28, 29].

## Conclusions

Disinfecting tonometer tips is clearly an important step in eye clinic care, but there is minimal morbidity caused by low-quality disinfection (dirty tonometry). Nosocomial eye infections do occur, but the only infection transmitted in eye clinics is EKC. Serious corneal infections, or systemic infections, which might lead to the loss of a QALY, have never been demonstrated to occur from tonometry despite billions of examinations. Therefore, the trade-offs between the time and costs of disinfection methods and the small differences in EKC risk are stark. Health systems cannot justify increased expenditure on interventions that do not save QALYs, and the cheapest, fastest method of alcohol wiping is clearly the justifiably sustainable choice.

Our review of the data highlighted that material compatibility is the primary concern of tonometer tip manufacturers in their disinfection guidance, and the data on microbiological effectiveness and cost effectiveness are lacking. Disinfection with alcohol, although possibly leading to accelerated degradation of the tip, is justified by the savings of time and money in the resource-constrained health system.

## Supplementary information

Appendix 1
