# Supplementary material for: Alcohol wipes are the most justifiably sustainable disinfection method for Goldmann tonometry
Source: Eye (Lond). 2025 May 20;39(10):1896–900. doi: 10.1038/s41433-025-03855-6 (PMC12209457; doi:10.1038/s41433-025-03855-6)
Supplement: Supplementary file 1 — Appendix 1 [file 41433_2025_3855_MOESM1_ESM.docx]

# Appendix 1

This table shows information on the microbiological effects of disinfectants, from external research where available (cited) and from the tonometer tip manufacturer’s recommendations and manuals for use (data not cited or published).

| **Disinfection Method** | **Active ingredient** | **Enveloped Virus** | **Non-enveloped virus (adenovirus)** | **Bacteria** | **Protozoa** | **Fungi** | **Prion** |
| --- | --- | --- | --- | --- | --- | --- | --- |
| Alcohols* | 70% Isopropyl alcohol | Yes < 1min^1^ | No | Yes^1^  (Tb 5min) | Acanthamoeba  trophoz. 30sec  cysts 5min^2^ | Yes < 1min^1^ | May increase adhesion to surfaces^3^ |
| * Note alcohols are not listed as a possible disinfectant by manufacturers of reusable tonometer tips, despite plenty of evidence about their disinfecting ability, partly due to concerns around damage to the PMMA material.^4-6^ Recent data refutes this concern about material compatability.^7^ | | | | | | | |
| Sodium Hypochlorite | Sodium hypochlorite (0.525%, 2.5%, 10%) | Yes 1 min^8^ | Yes 1 min^8^ | Yes | No Data | No Data | No Data |
| Hydrogen Peroxide | Hydrogen Peroxide (3%) | Yes | Conflicting^8^ | Yes 5 min^9^ | Yes 120 min^9^ | No Data | No Data |
| Acrylan | Quaternary Ammonium Compound  PHMB | Yes | No | No Data | No Data | No Data | No Data |
| Almyrol | PHMB  Quaternary Ammonium Compound | Yes 15min | No | Yes 15min  (Tb 30min) | No Data | No Data | No Data |
| Bodedex forte | Not available | Not available | Not available | Not available | Not available | Not available | Not available |
| Bomix plus | Quaternary ammonium compound | Yes | No | Yes 5min | No Data | No Data | No Data |
| Cidex OPA | 0.55% ortho-phthaldehyde | Yes 5min | Yes 1min^8^ | Yes 5min | No Data | Yes 5min | No Data |
| Dakin’s Solution | Sodium hypochlorite (**see above**) |  |  |  |  |  |  |
| Deconex 53 Instrument | Quaternary Ammonium Compound | Yes 5min | Yes 5min | Yes 5min | Not available | Yes 5min | No Data |
| Deconex 53 Plus | Guanidine Derivative  Quaternary ammonium compound | Yes 15min | Yes 30min | Yes 15min | No Data | Yes 15min | No Data |
| Endo Septol FF | Not available | Not available | Not available | Not available | Not available | Not available | Not available |
| Gigasept AF | Guanidine Derivative  Phenoxypropanol  Benzalkoniumchloride | Yes 60min | Yes 10min | Yes 15min | No Data | Yes 15min | No Data |
| Gigasept AF Forte | Quaternary Ammonium Compound  Phenoxypropanol  Guanidine Derivative  Laurylpropylene diamine | Yes 60min | Yes 5min | Yes 5min | No Data | Yes 5min | No Data |
| Gigasept FF New | Reaction product of DMO-THF, ethanol and water | Yes 15min | Yes 15min | Yes 15 min | No Data | Yes 60min | No Data |
| Gigasept PAA Concentrate | Peracetic acid | Yes 5min | Yes 5min | Yes 5 min | No Data | Yes 5min | No Data |
| Jiaen 6% Yoshida | Sodium hypochlorite 6% **(see above)** |  |  |  |  |  |  |
| Korsolex basic | Glutaraldehyde  (ethylendioxy)dimethanol | Yes 15min | Yes 15min | Yes 15min | No Data | Yes 15min | Not available |
| Korsolex extra | (Ethylendioxy)dimethanol  Glutaraldehyde  Quaternary Ammonium Compound | Yes 15min | Yes 15min | Yes 15min | No Data | Yes 15min | Not available |
| Mucocit-T | Quaternary Ammonium Compound  Laurylpropylene diamine | Yes 5min | No | Yes 30min | No Data | No Data | No Data |
| PeraSafe | Peracetic acid 1.62% | No Data | No Data | Yes 10min | No Data | No Data | No Data |
| Peraxylens | Not available | Not available | Not available | Not available | Not available | Not available | Not available |
| Perfekatan TB | Guanidine Derivative  Polar quaternary alkyl ammonium salt | Yes 15min | Yes 30min | Yes 15min | No Data | No Data | No Data |
| Perfekatan active | 1% Peracetic acid | Yes 15min | Yes 15min | Yes 5min | No Data | Yes 30min | No Data |
| Rapicide OPA/28 | Ortho-phthalaldehyde (0.575%) | Yes (some) | No data | Yes 5min | No Data | Yes (one)  5 min | No Data |
| Sekusept Aktiv | Peracetic acid | Yes 5 min | Yes 15 min | No data | No data | No data | No data |
| Sekusept forte S | Formaldehyde, Glyoxal, Glutaraldehyde  Benzylalkonium chloride | No data | No data | No data | No data | No data | No data |
| Sekusept PLUS | Glukoprotamine | Yes 15min | No data | Yes 15min | No data | No data | No data |
| Stabimed | Cocopropylendiamine | Yes 60min | Yes 5min | Yes 5min | No data | No data | No data |
| Sterihyde L | Glutaraldehyde | Not available | Not available | Not available | Not available | Not available | Not available |
| Tristel DUO OPH | Chlorine dioxide | Yes 30sec | Yes 30sec | Yes 30sec | No data | Yes 30sec | No data |

## References

1. Rutala WA, Weber DJ, Healthcare Infection Control Practices Advisory Committee (HICPAC). *Guideline for Disinfection and Sterilization in Healthcare Facilities*: Centres for Disease Control; 2019.

2. Aqeel Y, Rodriguez R, Chatterjee A, Ingalls RR, Samuelson J. Killing of diverse eye pathogens (Acanthamoeba spp., Fusarium solani, and Chlamydia trachomatis) with alcohols. *PLoS Negl Trop Dis* 2017; **11**(2)**:** e0005382.

3. Beekes M, Lemmer K, Thomzig A, Joncic M, Tintelnot K, Mielke M. Fast, broad-range disinfection of bacteria, fungi, viruses and prions. *J Gen Virol* 2010; **91**(Pt 2)**:** 580-589.

4. Key CB, Whitman J. Alcohol soaking damages applanation tonometer heads. *Arch Ophthalmol* 1986; **104**(6)**:** 800.

5. Kniestedt C, Sturmer J, Stamper RL. Clinical alert: damage to Goldmann applanation tonometer tips. *Acta Ophthalmol Scand* 2005; **83**(1)**:** 129-130.

6. Lingel NJ, Coffey B. Effects of disinfecting solutions recommended by the Centers for Disease Control on Goldmann tonometer biprisms. *J Am Optom Assoc* 1992; **63**(1)**:** 43-48.

7. Corbett J, Gale J. Effects of 70% Isopropyl Alcohol Disinfection on Goldmann Tonometer Tips. *Clin Exp Ophthalmol* 2025.

8. Rutala WA, Peacock JE, Gergen MF, Sobsey MD, Weber DJ. Efficacy of hospital germicides against adenovirus 8, a common cause of epidemic keratoconjunctivitis in health care facilities. *Antimicrob Agents Chemother* 2006; **50**(4)**:** 1419-1424.

9. Cillino S, Casuccio A, Giammanco GM, Mammina C, Morreale D, Di Pace F *et al.* Tonometers and infectious risk: myth or reality? Efficacy of different disinfection regimens on tonometer tips. *Eye (Lond)* 2007; **21**(4)**:** 541-546.
